# Benzoic acid–4-{(1*E*)-[(*E*)-2-(pyridin-4-yl­methyl­idene)hydrazin-1-yl­idene]meth­yl}pyridine (2/1)

**DOI:** 10.1107/S1600536810041875

**Published:** 2010-10-23

**Authors:** Hadi D. Arman, Trupta Kaulgud, Edward R. T. Tiekink

**Affiliations:** a Department of Chemistry, The University of Texas at San Antonio, One UTSA Circle, San Antonio, Texas 78249-0698, USA; b Department of Chemistry, University of Malaya, 50603 Kuala Lumpur, Malaysia

## Abstract

In the title co-crystal, C_12_H_10_N_4_·2C_7_H_6_O_2_, the complete 4-pyridine­aldazine mol­ecule is generated by a crystallographic centre of inversion. In the crystal, mol­ecules are connected into a three component aggregate *via* O—H⋯N hydrogen bonds. As both the benzoic acid [O—C—C—C torsion angle = 174.8 (2)°] and 4-pyridine­aldazine (r.m.s. deviation of the 16 non-H atoms = 0.041 Å) mol­ecules are almost planar, the resulting three-component aggregate is essentially planar. The crystal packing comprises layers of the three-component aggregates of alternating orientation stacked along the *b* axis; the connections between the mol­ecules are of the types C—H⋯π and π–π [pyridine–benzene centroid–centroid distance = 3.787 (4) Å].

## Related literature

For related studies on co-crystal formation involving the isomeric *n*-pyridine­aldazines, see: Broker *et al.* (2008 ▶); Arman *et al.* (2010*a* ▶,*b* ▶).
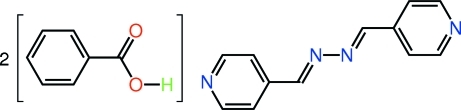


## Experimental

### 

#### Crystal data

C_12_H_10_N_4_·2C_7_H_6_O_2_
*M* *_r_* = 454.48
Monoclinic, 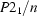

*a* = 6.873 (6) Å
*b* = 26.059 (19) Å
*c* = 7.117 (6) Å
β = 116.245 (13)°
*V* = 1143.3 (16) Å^3^
*Z* = 2
Mo *K*α radiation
μ = 0.09 mm^−1^
*T* = 98 K
0.40 × 0.26 × 0.08 mm

#### Data collection

Rigaku AFC12/SATURN724 diffractometer
6111 measured reflections
1956 independent reflections
1620 reflections with *I* > 2σ(*I*)
*R*_int_ = 0.063

#### Refinement

*R*[*F* ^2^ > 2σ(*F* ^2^)] = 0.072
*wR*(*F* ^2^) = 0.191
*S* = 1.12
1956 reflections
157 parameters
1 restraint
H atoms treated by a mixture of independent and constrained refinement
Δρ_max_ = 0.30 e Å^−3^
Δρ_min_ = −0.28 e Å^−3^

### 

Data collection: *CrystalClear* (Molecular Structure Corporation & Rigaku, 2005 ▶); cell refinement: *CrystalClear*; data reduction: *CrystalClear*; program(s) used to solve structure: *SHELXS97* (Sheldrick, 2008 ▶); program(s) used to refine structure: *SHELXL97* (Sheldrick, 2008 ▶); molecular graphics: *ORTEP-3* (Farrugia, 1997 ▶) and *DIAMOND* (Brandenburg, 2006 ▶); software used to prepare material for publication: *publCIF* (Westrip, 2010 ▶).

## Supplementary Material

Crystal structure: contains datablocks global, I. DOI: 10.1107/S1600536810041875/hb5684sup1.cif

Structure factors: contains datablocks I. DOI: 10.1107/S1600536810041875/hb5684Isup2.hkl

Additional supplementary materials:  crystallographic information; 3D view; checkCIF report

## Figures and Tables

**Table 1:** Hydrogen-bond geometry (Å, °) *Cg*1 is the centroid of the C2–C7 ring.

*D*—H⋯*A*	*D*—H	H⋯*A*	*D*⋯*A*	*D*—H⋯*A*
O1—H1*o*⋯N1^i^	0.85 (3)	1.80 (3)	2.642 (4)	175 (4)
C6—H6⋯*Cg*1^ii^	0.95	2.64	3.540 (5)	159

Symmetry codes: (i) 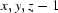; (ii) 
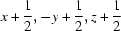
.

**Fig. 1.**
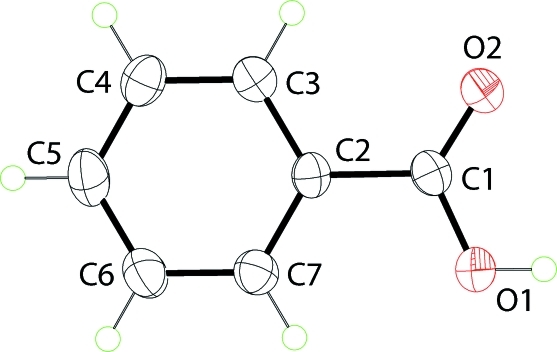
Molecular structure of benzoic acid found in the structure of (I) showing displacement ellipsoids at the 50% probability level

**Fig. 2.**
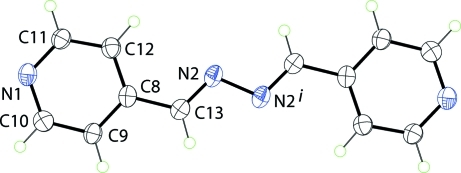
Molecular structure of 4-pyridinealdazine found in the structure of (I) showing displacement ellipsoids at the 50% probability level. The molecule is disposed about a centre of inversion with i = 1 - x, -y, 1 - z.

**Fig. 3.**
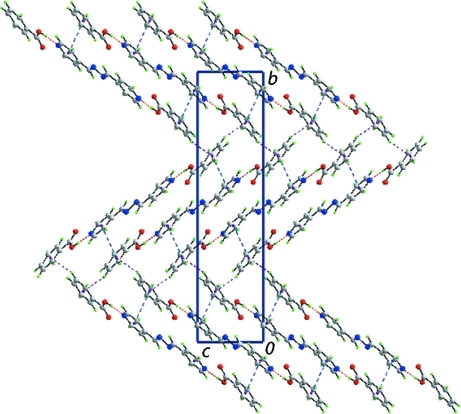
A view in projection down the a axis showing the stacking of layers comprising three component aggregates along b. The O—H···N, C—H···π and π–π interactions are shown as orange, purple and blue dashed lines, respectively.

## supplementary crystallographic information

### Comment

In connection with co-crystallization studies of the isomeric n-pyridinealdazines (Broker *et al.*, 2008; Arman *et al.*, 2010*a*,*b*), the co-crystallization of benzoic acid and 4-pyridinealdazine was investigated. This lead to the isolation of the title 2/1 co-crystal, (I).

The asymmetric unit in (I) comprises a molecule of benzoic acid, Fig. 1, and half a molecule of 4-pyridinealdazine, with the latter disposed about a centre of inversion, Fig. 2. The constituents of (I) are connected by O—H···N hydrogen bonds, Table 1, to generate a centrosymmetric three component aggregate. The carboxylic acid group is co-planar with the benzene ring to which it is connected [the O1—C1—C2—C3 torsion angle is 174.8 (2) °] and, similarly, the 4-pyridinealdazine molecule is planar with the r.m.s. deviation of the 16 non-hydrogen atoms being 0.041 Å [maximum deviation = 0.075 (3) Å for the methylene-C13 atom]. Accordingly, the three component aggregate is essentially planar.

In the crystal packing, layers of three component aggregates of alternating orientation stack along the *b* axis, Fig. 3. Connections between the molecules are of the type C—H···π, Table 1, and π–π [ring centroid(N1,C8–C12)···ring centroid(C2–C7) = 3.787 (4) Å].

### Experimental

Yellow blocks of (I) were isolated from the 2/1 co-crystallization of 2-phenylacetic acid (Sigma Aldrich) and 4-[(1*E*)-[(*E*)-2-(pyridin-4-ylmethylidene)hydrazin-1-ylidene]methyl]pyridine(Sigma Aldrich), in ethanol; m. pt. 395–397 K.

IR assignment (cm^-1^): 2923 ν(C—H); 1693 ν(C═O); 1602 ν(C═N); 1492, 1453, 1409 ν(C—C(aromatic)); 1306 ν(C—N); 817, 716 δ(C—H).

### Refinement

C-bound H-atoms were placed in calculated positions (C–H 0.95 Å) and were included in the refinement in the riding model approximation with *U*_iso_(H) set to 1.2*U*_eq_(C). The O-bound H-atom was located in a difference Fourier map and was refined with a distance restraint of O–H 0.84±0.01 Å, and with *U*_iso_(H) = 1.5*U*_eq_(O).

### Crystal data

**Table:**

C_12_H_10_N_4_·2C_7_H_6_O_2_	*F*(000) = 476
*M**_r_* = 454.48	*D*_x_ = 1.320 Mg m^−^^3^
Monoclinic, *P*2_1_/*n*	Mo *K*α radiation, λ = 0.71073 Å
Hall symbol: -P 2yn	Cell parameters from 4161 reflections
*a* = 6.873 (6) Å	θ = 3.3–40.2°
*b* = 26.059 (19) Å	µ = 0.09 mm^−^^1^
*c* = 7.117 (6) Å	*T* = 98 K
β = 116.245 (13)°	Block, yellow
*V* = 1143.3 (16) Å^3^	0.40 × 0.26 × 0.08 mm
*Z* = 2	

### Data collection

**Table:**

Rigaku AFC12K/SATURN724 diffractometer	1620 reflections with *I* > 2σ(*I*)
Radiation source: fine-focus sealed tube	*R*_int_ = 0.063
graphite	θ_max_ = 25.0°, θ_min_ = 3.1°
ω scans	*h* = −8→8
6111 measured reflections	*k* = −30→30
1956 independent reflections	*l* = −8→6

### Refinement

**Table:**

Refinement on *F*^2^	Primary atom site location: structure-invariant direct methods
Least-squares matrix: full	Secondary atom site location: difference Fourier map
*R*[*F*^2^ > 2σ(*F*^2^)] = 0.072	Hydrogen site location: inferred from neighbouring sites
*wR*(*F*^2^) = 0.191	H atoms treated by a mixture of independent and constrained refinement
*S* = 1.12	*w* = 1/[σ^2^(*F*_o_^2^) + (0.083*P*)^2^ + 0.7947*P*] where *P* = (*F*_o_^2^ + 2*F*_c_^2^)/3
1956 reflections	(Δ/σ)_max_ < 0.001
157 parameters	Δρ_max_ = 0.30 e Å^−^^3^
1 restraint	Δρ_min_ = −0.28 e Å^−^^3^

### Special details

**Table:**

Geometry. All s.u.'s (except the s.u. in the dihedral angle between two l.s. planes) are estimated using the full covariance matrix. The cell s.u.'s are taken into account individually in the estimation of s.u.'s in distances, angles and torsion angles; correlations between s.u.'s in cell parameters are only used when they are defined by crystal symmetry. An approximate (isotropic) treatment of cell s.u.'s is used for estimating s.u.'s involving l.s. planes.
Refinement. Refinement of *F*^2^ against ALL reflections. The weighted *R*-factor *wR* and goodness of fit *S* are based on *F*^2^, conventional *R*-factors *R* are based on *F*, with *F* set to zero for negative *F*^2^. The threshold expression of *F*^2^ > 2σ(*F*^2^) is used only for calculating *R*-factors(gt) *etc*. and is not relevant to the choice of reflections for refinement. *R*-factors based on *F*^2^ are statistically about twice as large as those based on *F*, and *R*- factors based on ALL data will be even larger.

### Fractional atomic coordinates and isotropic or equivalent isotropic displacement parameters (Å^2^)

**Table:**

	*x*	*y*	*z*	*U*_iso_*/*U*_eq_	
O1	0.2366 (3)	0.14824 (8)	0.4031 (3)	0.0367 (5)	
H1o	0.254 (6)	0.1339 (13)	0.305 (4)	0.055*	
O2	−0.0291 (3)	0.09135 (7)	0.3447 (3)	0.0348 (5)	
C1	0.0732 (4)	0.12953 (10)	0.4330 (4)	0.0279 (6)	
C2	0.0247 (4)	0.15989 (10)	0.5862 (4)	0.0272 (6)	
C3	−0.1544 (5)	0.14691 (10)	0.6194 (4)	0.0294 (6)	
H3	−0.2428	0.1185	0.5480	0.035*	
C4	−0.2032 (5)	0.17572 (11)	0.7573 (4)	0.0363 (7)	
H4	−0.3265	0.1673	0.7784	0.044*	
C5	−0.0723 (6)	0.21684 (12)	0.8644 (5)	0.0422 (8)	
H5	−0.1061	0.2363	0.9591	0.051*	
C6	0.1078 (6)	0.22960 (12)	0.8335 (5)	0.0415 (8)	
H6	0.1974	0.2576	0.9074	0.050*	
C7	0.1563 (5)	0.20132 (11)	0.6942 (4)	0.0334 (7)	
H7	0.2788	0.2101	0.6723	0.040*	
N1	0.2915 (4)	0.09852 (9)	1.1065 (3)	0.0308 (6)	
N2	0.5060 (4)	0.01762 (9)	0.5783 (3)	0.0316 (6)	
C8	0.3397 (4)	0.03986 (10)	0.8005 (4)	0.0268 (6)	
C9	0.1647 (4)	0.03201 (10)	0.8464 (4)	0.0285 (6)	
H9	0.0594	0.0065	0.7743	0.034*	
C10	0.1466 (5)	0.06209 (10)	0.9991 (4)	0.0312 (7)	
H10	0.0264	0.0567	1.0288	0.037*	
C11	0.4597 (4)	0.10580 (11)	1.0616 (4)	0.0293 (6)	
H11	0.5632	0.1315	1.1365	0.035*	
C12	0.4898 (5)	0.07771 (10)	0.9114 (4)	0.0297 (6)	
H12	0.6110	0.0842	0.8843	0.036*	
C13	0.3593 (4)	0.00791 (10)	0.6385 (4)	0.0278 (6)	
H13	0.2627	−0.0200	0.5783	0.033*	

### Atomic displacement parameters (Å^2^)

**Table:**

	*U*^11^	*U*^22^	*U*^33^	*U*^12^	*U*^13^	*U*^23^
O1	0.0403 (12)	0.0415 (12)	0.0340 (12)	−0.0088 (9)	0.0215 (9)	−0.0103 (9)
O2	0.0347 (11)	0.0351 (11)	0.0342 (11)	−0.0036 (9)	0.0150 (9)	−0.0074 (8)
C1	0.0293 (14)	0.0281 (14)	0.0218 (14)	0.0032 (11)	0.0073 (11)	0.0008 (10)
C2	0.0322 (14)	0.0282 (14)	0.0206 (13)	0.0035 (11)	0.0112 (11)	0.0028 (10)
C3	0.0353 (15)	0.0253 (14)	0.0256 (14)	0.0018 (11)	0.0116 (11)	0.0043 (11)
C4	0.0401 (17)	0.0392 (16)	0.0346 (16)	0.0054 (13)	0.0210 (13)	0.0058 (12)
C5	0.059 (2)	0.0381 (17)	0.0346 (17)	0.0030 (15)	0.0255 (15)	−0.0042 (13)
C6	0.0503 (19)	0.0390 (17)	0.0370 (17)	−0.0072 (14)	0.0210 (14)	−0.0098 (13)
C7	0.0371 (16)	0.0337 (16)	0.0293 (15)	−0.0027 (12)	0.0145 (12)	−0.0005 (11)
N1	0.0353 (13)	0.0328 (13)	0.0241 (12)	0.0048 (10)	0.0130 (10)	0.0024 (9)
N2	0.0405 (14)	0.0303 (13)	0.0235 (12)	0.0005 (10)	0.0138 (11)	−0.0029 (9)
C8	0.0301 (14)	0.0278 (14)	0.0211 (13)	0.0076 (11)	0.0100 (11)	0.0044 (10)
C9	0.0313 (14)	0.0321 (14)	0.0198 (13)	0.0020 (11)	0.0091 (11)	0.0013 (10)
C10	0.0295 (15)	0.0341 (15)	0.0286 (15)	0.0027 (11)	0.0115 (12)	0.0031 (11)
C11	0.0300 (14)	0.0302 (14)	0.0250 (14)	0.0018 (11)	0.0098 (11)	0.0002 (10)
C12	0.0298 (14)	0.0323 (15)	0.0263 (14)	0.0024 (11)	0.0118 (12)	0.0011 (11)
C13	0.0328 (14)	0.0246 (13)	0.0234 (14)	0.0025 (11)	0.0100 (11)	0.0022 (10)

### Geometric parameters (Å, °)

**Table:**

O1—C1	1.325 (3)	N1—C11	1.342 (4)
O1—H1O	0.85 (3)	N1—C10	1.343 (4)
O2—C1	1.220 (3)	N2—C13	1.283 (4)
C1—C2	1.499 (4)	N2—N2^i^	1.418 (4)
C2—C3	1.395 (4)	C8—C12	1.393 (4)
C2—C7	1.400 (4)	C8—C9	1.394 (4)
C3—C4	1.389 (4)	C8—C13	1.476 (4)
C3—H3	0.9500	C9—C10	1.390 (4)
C4—C5	1.390 (4)	C9—H9	0.9500
C4—H4	0.9500	C10—H10	0.9500
C5—C6	1.390 (5)	C11—C12	1.384 (4)
C5—H5	0.9500	C11—H11	0.9500
C6—C7	1.389 (4)	C12—H12	0.9500
C6—H6	0.9500	C13—H13	0.9500
C7—H7	0.9500		
			
C1—O1—H1o	114 (3)	C2—C7—H7	120.0
O2—C1—O1	123.6 (3)	C11—N1—C10	117.7 (2)
O2—C1—C2	122.8 (3)	C13—N2—N2^i^	110.7 (3)
O1—C1—C2	113.6 (2)	C12—C8—C9	118.1 (2)
C3—C2—C7	119.9 (3)	C12—C8—C13	122.8 (3)
C3—C2—C1	119.5 (2)	C9—C8—C13	119.1 (2)
C7—C2—C1	120.6 (3)	C10—C9—C8	119.0 (3)
C4—C3—C2	119.7 (3)	C10—C9—H9	120.5
C4—C3—H3	120.1	C8—C9—H9	120.5
C2—C3—H3	120.1	N1—C10—C9	122.9 (3)
C3—C4—C5	120.3 (3)	N1—C10—H10	118.5
C3—C4—H4	119.8	C9—C10—H10	118.5
C5—C4—H4	119.8	N1—C11—C12	123.2 (3)
C6—C5—C4	120.2 (3)	N1—C11—H11	118.4
C6—C5—H5	119.9	C12—C11—H11	118.4
C4—C5—H5	119.9	C11—C12—C8	119.1 (3)
C5—C6—C7	119.8 (3)	C11—C12—H12	120.5
C5—C6—H6	120.1	C8—C12—H12	120.5
C7—C6—H6	120.1	N2—C13—C8	120.4 (2)
C6—C7—C2	120.1 (3)	N2—C13—H13	119.8
C6—C7—H7	120.0	C8—C13—H13	119.8
			
O2—C1—C2—C3	−5.1 (4)	C12—C8—C9—C10	0.2 (4)
O1—C1—C2—C3	174.8 (2)	C13—C8—C9—C10	180.0 (2)
O2—C1—C2—C7	175.5 (2)	C11—N1—C10—C9	0.3 (4)
O1—C1—C2—C7	−4.6 (4)	C8—C9—C10—N1	−0.4 (4)
C7—C2—C3—C4	1.0 (4)	C10—N1—C11—C12	0.0 (4)
C1—C2—C3—C4	−178.4 (2)	N1—C11—C12—C8	−0.1 (4)
C2—C3—C4—C5	−1.0 (4)	C9—C8—C12—C11	0.1 (4)
C3—C4—C5—C6	0.3 (4)	C13—C8—C12—C11	−179.7 (2)
C4—C5—C6—C7	0.3 (5)	N2^i^—N2—C13—C8	−179.7 (2)
C5—C6—C7—C2	−0.3 (5)	C12—C8—C13—N2	−7.2 (4)
C3—C2—C7—C6	−0.3 (4)	C9—C8—C13—N2	173.0 (2)
C1—C2—C7—C6	179.1 (2)		

Symmetry codes: (i) −*x*+1, −*y*, −*z*+1.

### Hydrogen-bond geometry (Å, °)

**Table:**

Cg1 is the centroid of the C2–C7 ring.

**Table:**

*D*—H···*A*	*D*—H	H···*A*	*D*···*A*	*D*—H···*A*
O1—H1o···N1^ii^	0.85 (3)	1.80 (3)	2.642 (4)	175 (4)
C6—H6···Cg1^iii^	0.95	2.64	3.540 (5)	159

Symmetry codes: (ii) *x*, *y*, *z*−1; (iii) *x*+1/2, −*y*+1/2, *z*+1/2.
